# Neutrophilia, lymphopenia and myeloid dysfunction: a living review of the quantitative changes to innate and adaptive immune cells which define COVID-19 pathology

**DOI:** 10.1093/oxfimm/iqab016

**Published:** 2021-07-15

**Authors:** Amy S Codd, Stephanie J Hanna, Ewoud B Compeer, Felix C Richter, Eleanor J Pring, Ester Gea-Mallorquí, Mariana Borsa, Owen R Moon, D Oliver Scourfield, Oxford-Cardiff COVID-19 Literature Consortium, David J Ahern, Hannah Almuttaqi, Dominic S Alonzi, Aljawharah Alrubayyi, Ghada Alsaleh, Valentina M T Bart, Vicky Batchelor, Rebecca Bayliss, Dorothée L Berthold, Jelena S Bezbradica, Tehmina Bharuchq, Helene Borrmann, Mariana Borsa, Rowie Borst, Juliane Brun, Stephanie Burnell, Lorenzo Capitani, Athena Cavounidis, Lucy Chapman, Anne Chauveau, Liliana Cifuentes, Amy Susan Codd, Ewoud Bernardus Compeer, Clarissa Coveney, Amy Cross, Sara Danielli, Luke C Davies, Calliope A Dendrou, Sandra Dimonte, Ruban Rex Peter Durairaj, Lynn B Dustin, Arthur Dyer, Ceri Fielding, Fabian Fischer, Awen Gallimore, Sarah Galloway, Anís Gammage, Ester Gea-Mallorquí, Andrew Godkin, Stephanie Jean Hanna, Cornelia Heuberger, Sarah Hulin-Curtis, Fadi Issa, Emma Jones, Ruth Jones, Kristin Ladell, Sarah N Lauder, Kate Liddiard, Petros Ligoxygakis, Fangfang Lu, Bruce MacLachlan, Shayda Maleki-Toyserkani, Elizabeth H Mann, Anna M Marzeda, Reginald James Matthews, Julie M Mazet, Anita Milicic, Emma Mitchell, Owen Moon, Van Dien Nguyen, Miriam O'Hanlon, Clara Eléonore Pavillet, Dimitra Peppa, Ana Pires, Eleanor Pring, Max Quastel, Sophie Reed, Jan Rehwinkel, Niamh Richmond, Felix Clemens Richter, Alice J B Robinson, Patrícia R S Rodrigues, Pragati Sabberwal, Arvind Sami, Raphael Sanches Peres, Quentin Sattentau, Barbora Schonfeldova, David Oliver Scourfield, Tharini A Selvakumar, Freya R Shepherd, Cariad Shorten, Anna Katharina Simon, Adrian L Smith, Alicia Teijeira Crespo, Michael Tellier, Emily Thornton, Lion F K Uhl, Erinke van Grinsven, Angus K T Wann, Richard Williams, Joseph D Wilson, Dingxi Zhou, Zihan Zhu, Awen M Gallimore, Anita Milicic

**Affiliations:** 1 Division of Infection and Immunity, School of Medicine, Cardiff University, Cardiff, UK; 2 Nuffield Department of Orthopaedics, Rheumatology and Musculoskeletal Sciences, Kennedy Institute of Rheumatology, University of Oxford, Oxford, UK; 3 Viral Immunology Unit, Nuffield Department of Medicine, University of Oxford, Oxford, UK; 4 Nuffield Department of Medicine, The Jenner Institute, University of Oxford, Oxford, UK

**Keywords:** SARS-CoV-2, lymphocytes, lymphopenia, neutrophils, neutrophilia, monocytes, B cells, severity, recovery, cell counts, clinical, prognosis

## Abstract

Destabilization of balanced immune cell numbers and frequencies is a common feature of viral infections. This occurs due to, and further enhances, viral immune evasion and survival. Since the discovery of the Severe Acute Respiratory Syndrome coronavirus 2 (SARS-CoV-2), which manifests in coronavirus disease 2019 (COVID-19), a great number of studies have described the association between this virus and pathologically increased or decreased immune cell counts. In this review, we consider the absolute and relative changes to innate and adaptive immune cell numbers, in COVID-19. In severe disease particularly, neutrophils are increased, which can lead to inflammation and tissue damage. Dysregulation of other granulocytes, basophils and eosinophils represents an unusual COVID-19 phenomenon. Contrastingly, the impact on the different types of monocytes leans more strongly to an altered phenotype, e.g. HLA-DR expression, rather than numerical changes. However, it is the adaptive immune response that bears the most profound impact of SARS-CoV-2 infection. T cell lymphopenia correlates with increased risk of intensive care unit admission and death; therefore, this parameter is particularly important for clinical decision-making. Mild and severe diseases differ in the rate of immune cell counts returning to normal levels post disease. Tracking the recovery trajectories of various immune cell counts may also have implications for long-term COVID-19 monitoring. This review represents a snapshot of our current knowledge, showing that much has been achieved in a short period of time. Alterations in counts of distinct immune cells represent an accessible metric to inform patient care decisions or predict disease outcomes.

## INTRODUCTION

Coronavirus disease 2019 (COVID-19) is a global pandemic caused by infection with Severe Acute Respiratory Syndrome Coronavirus 2 (SARS-CoV-2). Since the outbreak, it has become apparent that there is a broad spectrum of clinical symptoms in people infected with SARS-CoV-2: from no obvious symptoms in around 40% of infected individuals [1] to a need for intensive care unit (ICU) hospitalization and use of ventilators in most severely affected patients [2]. Additionally, the exacerbated immune response contributes to ‘acute respiratory distress syndrome’ (ARDS), which is a prominent feature of severe COVID-19 [3, 4]. Many studies have reported singular and additive epidemiological and clinical risk factors associated with increased COVID-19 severity and mortality, including age and gender, and pre-existing conditions such as obesity, diabetes, hypertension and cardiovascular disease [2, 5, 6]. Ethnicity is a complex etiological feature when considering the impact of COVID-19. In addition to some of the abovementioned co-morbidities, ethnicity impacts socio-economic status, access to healthcare and occupational hazard. Ethnicity was inconsistently reported in the early stages of the pandemic [7], although the weight of evidence recently identified a significantly increased risk of COVID-19 infection in Black and Asian, compared to White individuals [8]. Data from the USA show that the mortality rate from COVID-19 is also higher in Black compared to white ethnic groups [9].

In this review, we summarize the current knowledge of the changes observed in absolute counts and phenotypic frequencies of immune cells in SARS-CoV-2-infected individuals. We try to understand the nature of the immune response that leads to recovery over severe disease and how treatments can help to promote an immune response that leads to recovery. For hospitalized COVID-19 patients, rapid measures to guide stratification of care and resources are crucial, due to the burden of the pandemic on healthcare systems. A single centre evaluation of ‘core’ (full blood counts, urea, electrolytes, liver function and C-reactive protein) versus ‘extended’ (D-dimer, ferritin, high-sensitivity troponin I, lactate dehydrogenase, procalcitonin) clinical tests found the latter did not add sufficient cost-benefit prognostic value [10]. As a routine readout, cell counts provide a valuable overview of the main cell types involved in the immune response to COVID-19. Cell counts are informative even in the absence of mechanistic information explaining increased or decreased numbers, and thus could be used to guide clinical decision-making and signpost more in-depth, descriptive research such as multi-dimensional phenotyping and biomarker identification.

## WHITE BLOOD CELL COUNT

As yet there is no clear trend in overall white blood cell (WBC) count in individuals infected with SARS-CoV-2; a systematic review found increased WBC in 24.26% and decreased WBC in 10.55% in 20 662 hospitalized COVID-19 patients [11]. However, meta-analysis of 45 studies covering such patients showed a trend between increased WBC count and disease severity [12]. This corroborates earlier observations of elevated WBC counts in severe disease and deceased COVID-19 patients [13–16] and ICU admission [17].

**Figure 1:**
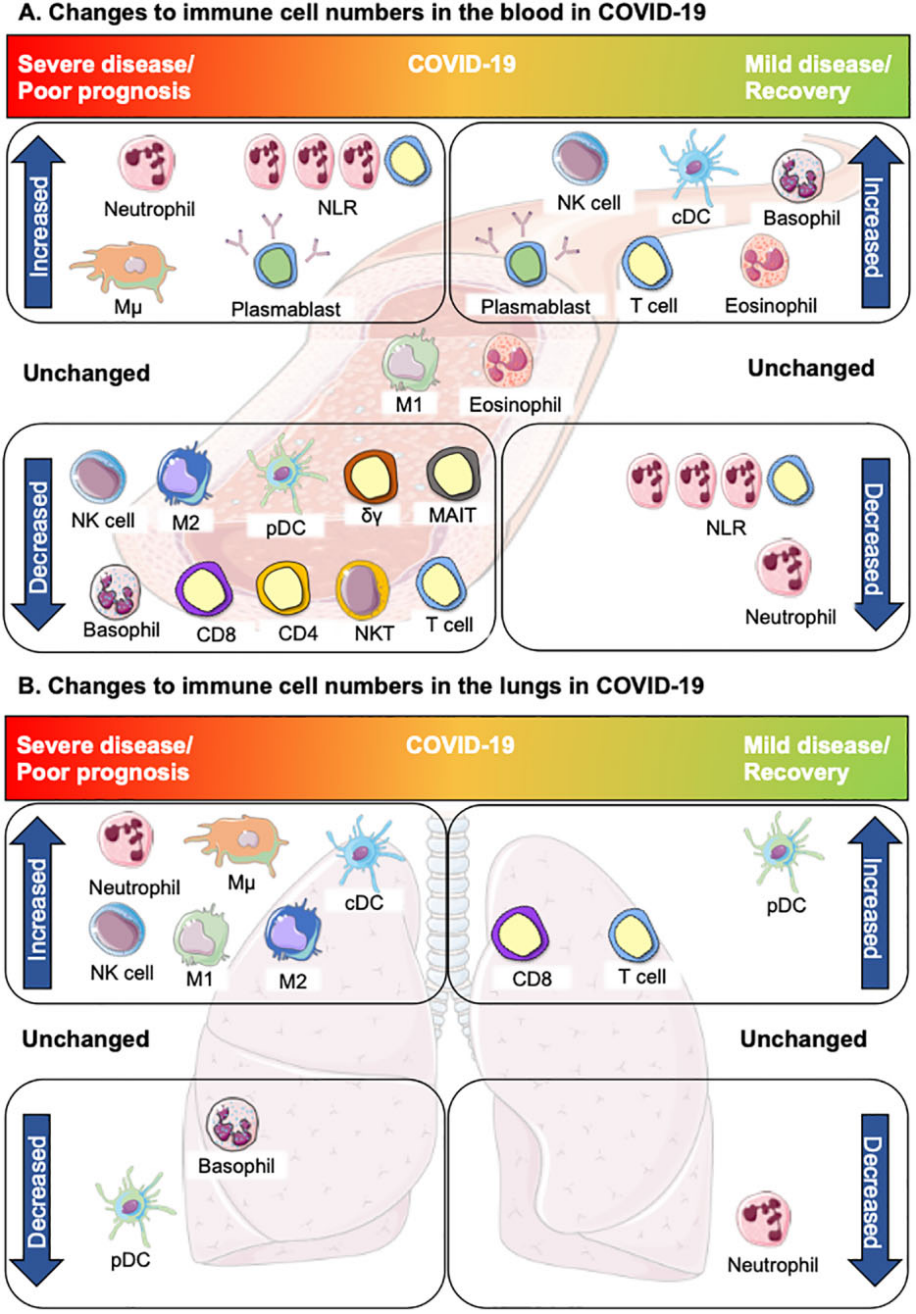
Changes in innate and adaptive immune cell numbers in severe and mild/recovered COVID-19. (**a**) Altered immune cells in the peripheral blood. Top left, severe disease: neutrophils, inflammatory intermediate monocytes (Mµ) and the NLR are increased in severe COVID-19. Increased plasmablasts have been reported in both severe and mild disease. Bottom left: Many cells of the innate and adaptive immune system are depleted in severe COVID-19: non-classical monocytes (M2), pDC and basophils. Lymphopenia is widely reported in COVID-19, largely due to depletion of T cells; reductions in CD4^+^ and/or CD8^+^ T cells (CD4, CD8) have been reported and innate lymphocytes and unconventional T cells are also decreased: NK cells, NKT cells, γδ T cells and MAIT cells. Centre, unchanged: some studies suggest eosinophils are unchanged in COVID-19 disease but may increase in the course of recovery. Classical monocytes (M1) are largely unchanged in COVID-19, however, changes in expression of certain phenotypic proteins are associated with severe disease. Top right, mild COVID-19/recovery: Increasing basophils and eosinophils are also associated with recovery or mild COVID-19, along with higher numbers of cDCs, NK cells and T cells which are indicative of an effective anti-viral immune response contributing to a milder form of COVID-19 and/or recovery. Bottom left: decreased neutrophils and recovery of lymphocyte numbers, resulting in a reduction of the NLR, are associated with recovery from COVID-19. (**b**) Altered immune cells in the lungs. Top left: Severe disease is largely characterized by inflammation in the lungs in association with increased neutrophils, M1 and Mµ monocytes. NK cells, cDCs and M2 monocytes are also increased, whereas basophils are decreased. Despite decreases in the blood, pDCs are rarely detected in the lung in severe COVID-19. In mild disease or recovery, neutrophils return to normal ranges. Increased T cells, in particular CD8^+^ T cells, occur as part of the recovery from COVID-19, although some studies have reported delayed recovery trajectories based on disease severity. DCs also increase with recovery. This figure was created using Servier Medical Art templates, which are licensed under a Creative Commons Attribution 3.0 Unported License; https://smart.servier.com.

Recent studies might explain the lack of a strong correlation between WBC count and disease severity, as an increase in the neutrophil to lymphocyte ratio (NLR), characterized by reduced lymphocytes and elevated neutrophils, has been found in patients with normal WBC counts upon hospital admission [18–20]. These non-convergent data demonstrate a variety of immune cell perturbations across the spectrum of COVID-19 severity (Fig. 1), necessitating the examination of specific immune cell subsets.

## INNATE IMMUNE CELLS IN SARS-COV-2 INFECTION

Neutrophils are the most prominent innate cells in the response to a viral infection and have been widely reported to be increased in the blood in COVID-19 patients [15, 17, 21]. Neutrophil infiltration of pulmonary capillaries has also been described, along with increased expression of neutrophil-associated chemokines in lung epithelial cells [22, 23]. Neutrophilia appears to be self-propelled by initial viral evasion of immune detection, leading to poor viral clearance, resulting in inflammation and cytokine storm [24–27]. There are limited data providing greater detail about neutrophil subsets in COVID-19, although Kuri-Cervantes *et al.* and Wilk *et al.* describe characteristics of neutrophils that may suggest impaired maturation (reduced CD15 expression and expression of developmental markers specifically in ARDS patients, respectively) [28, 29]. Atypical neutrophil phenotypes have been associated with excessive Neutrophil Extracellular Trap (NET) production, which can cause hyperinflammation and tissue damage [24, 30].

Increased numbers of peripheral neutrophils correlate with COVID-19 disease severity and poor outcome [15, 17, 31–33]. The contribution of dysfunctional neutrophil anti-viral responses to COVID-19 pathology is discussed in further detail in the innate immune response-focused article of our living review series [34]. The relationship between neutrophil and lymphocyte counts (NLR) represents a more powerful prognostic measure, both in COVID-19 and other acute disease settings [15, 35, 36] and is discussed in detail in the adaptive immunity section of this review.

Basophils and eosinophils, which play a greater role in other innate immune functions, such as allergic and anti-microbial responses, are nevertheless also impacted in COVID-19. The relatively limited data indicate depletion of basophils occurs in the blood in COVID-19, showing some associations with severe disease [15, 37–40]. It has been suggested, however, that decreased basophil counts in the blood could be attributed to migration to the lungs [37, 41]. While the cause of basophil depletion is currently unknown, a basophil count of 25/µl in the blood may represent a threshold predictive of survival in ventilated patients [42]. The directionality of eosinophil perturbation in COVID-19 is less clear; several studies report decreased or unchanged eosinophil numbers in the blood [15, 37, 39, 40, 43, 44]. Contrastingly, however, Lucas *et al.* describe a sustained increase in peripheral eosinophils in severe disease, along with increased IL-5, which is a contributing signalling factor for eosinopoiesis in the bone marrow [45, 46]. Increased IL-5 has been reported in other studies, however, without eosinophil counts [6, 47]. The limited data available on lung eosinophil infiltration are also conflicted [48–50], although it is not clear if a distinction was made for the detection of migratory or lung-resident eosinophils. Whilst stratifying patients by peripheral blood eosinophil counts (> or < 0.02 × 10^9^/L) has revealed differences in severity and mortality [51, 52], overall, this suggests a need for more in-depth investigation of IL-5 associated eosinophil responses in the blood and potentially in the lungs.

In terms of absolute numbers, studies reported unchanged [20, 28, 38, 39], increased [14, 33, 45, 53, 54] or decreased frequencies of the monocytic cell lineage in peripheral blood during COVID-19 [15, 29, 55, 56]. Some of this discrepancy is likely due to the use of different markers and nomenclature in the studies: reporting global increases or decreases in monocytes is unlikely to capture their significance in the context of COVID-19 symptoms or clinical course, due to phenotypically and functionally distinct monocyte subsets. However, some trends have emerged. In peripheral blood, classical monocytes (M1; CD14^+^ CD16^−^) were within the reference range in early disease [20] and remained stable in severe or moderate disease [45]. Sánchez-Cerrillo *et al.* found that the frequency (rather than absolute count) of classical monocytes was reduced in the periphery, and enriched, although relatively infrequent, in bronchoscopy samples of patients with severe COVID-19 [56]. Reduction in peripheral blood non-classical (M2; CD14^−^ CD16^+^) monocytes has been reported in severe and moderate disease and in ARDS compared to controls, although similarly this might be associated with a migration to the lung, due to the observed enrichment in bronchoscopy samples [45, 56, 57]. The most prominently reported monocytic perturbation is the expansion of intermediate (Mµ; CD14^+^ CD16^+^) monocytes in peripheral blood [37, 38, 57, 58].

The significance of these changes in monocyte numbers is not yet clear. IL-6 production by intermediate monocytes in COVID-19 has been described in association with cytokine storm and severe disease [59], and in general, increased IL-6 levels correlate with disease severity [2, 60]. However, since an increase in intermediate monocytes in blood has been reported in both mild and severe disease, it is likely that other immune cells contribute to IL-6 production [28, 37, 38, 56, 57, 59]. The ability of SARS-CoV-2 to infect monocytes has been demonstrated *in vitro*, using primary monocytes and monocyte cell lines [5, 55], resulting in pro- and anti-inflammatory cytokine production, including interferon (IFN) α, β and λ, and tumour necrosis factor (TNF), IL-1β, IL-6, IL-10 and transforming growth factor (TGF)β [5, 55]. Cytokine and chemokine production by monocytes in COVID-19 has recently been reviewed by our consortium [34]. Some studies have also identified upregulation and co-expression of M1 and M2 markers on monocytes [61], which further complicates efforts to understand the contribution of functional monocyte subsets in COVID-19. Boumaza *et al.* found that polarized monocyte cell lines (M1 or M2) showed no difference in the propensity for *in vitro* SARS-CoV-2 infection, however, infection led to the general upregulation of M2 markers [55]. Further work is therefore required to establish a clear picture of the contributing role of the various monocyte subsets in COVID-19.

Overall dendritic cell (DC) numbers are reduced in the peripheral blood in COVID-19 [3, 28, 29, 37, 62]. In humans, DCs are generally divided into conventional DC subsets (cDCs), specialized for antigen presentation, and plasmacytoid DC (pDCs) which primarily produce Type 1 IFN and are important for anti-viral response [63]. In COVID-19, pDCs appear preferentially depleted [38, 53, 64]. Lower pDC numbers in the periphery are reported to correlate with severe disease [20, 29]. Additionally, Sanchez-Cerillo *et al.* did not detect pDCs in bronchiolar samples, although it is unclear if this was due to impaired migration or depletion in the periphery [56].

In conclusion, the available data on the innate response to SARS-CoV-2 infection indicate an association between the efficacy of the early granulocyte (eosinophil, basophil and neutrophil) response and disease severity. It has been suggested that SARS-CoV-2 can evade immune sensing and inhibit signalling [34, 65–68], resulting in impaired activation of the innate response. Quantifying monocytes in COVID-19 is particularly complex as the classical, non-classical and intermediate subtypes function at various stages of the immune response, including its resolution. Conclusions thus far are largely reliant on data pertaining to peripheral blood monocytes, which overlook, and could underestimate, the impact of monocyte migration to the lungs. While not reviewed here, it has also been suggested that altered expression of certain markers, e.g. HLA-DR (decreased), on monocytes and other cell types, is characteristic in COVID-19 [20, 28, 29, 37, 45, 55, 61, 69]. Laing *et al.* propose a COVID-19 innate and adaptive immune signature which is largely in agreement with the quantitative data discussed here: depletion of pDCs and basophils correlate with COVID-19 severity, while alterations in the proportion of different monocyte lineages demarcate COVID-19 from other respiratory infections [37].

## LYMPHOPENIA AND THE ADAPTIVE IMMUNE RESPONSE TO SARS-COV-2

Reduction in lymphocytes, known as lymphopenia, is a common, although not exclusive, characteristic of COVID-19. Lymphopenia also occurs in infections with Ebola virus, respiratory syncytial virus (RSV)—which most commonly affects children, SARS-CoV-1, Middle Eastern Respiratory Virus (MERS) and Human Immunodeficiency Virus (HIV). It has also been reported for some strains of Influenza [70–74]. In COVID-19, lymphopenia most prominently affects T cells, as discussed below; quantitative changes to NK cells, and, less commonly, B cells, have also been reported [15, 18, 20, 28, 37, 38, 57, 75–78].

## NK CELLS

NK cells are innate lymphocytes important in early viral infection control through direct killing of infected cells, by lysis or antibody-directed cellular cytotoxicity, and production of cytokines such as IFNγ [79, 80]. NK cell depletion in peripheral blood has been reported as a part of COVID-19-associated lymphopenia [20], although it has been noted that this is not as extensive as T cell lymphopenia [37]. Maucourant *et al.* further found that the absolute counts of both CD56^dim^ (cytotoxic and IFNγ^+^) and CD56^bright^ (cytokine-producing and IFNγ^+^) NK cells were reduced [81]. This reduction positively correlates with COVID-19 severity, ICU admission and increased hospital stay [15, 38, 57, 75, 76, 78, 82–84]. Carsetti *et al.* and Odak *et al.* observed that an increase in NK cells was associated with asymptomatic infection, and mild COVID-19 patients had NK cell numbers comparable to healthy controls, respectively [57, 83]. In addition to reduced NK cell numbers, Mazzoni *et al*. found that IL-6 levels negatively correlate with NK cell cytotoxic capacity (granzyme production) [39], suggesting a mechanism for poor infection control. Preservation of NK cell numbers and function in the periphery is therefore an important factor in efficient COVID-19 infection control. The mechanisms by which NK cells could control SARS-CoV-2 infection have recently been discussed by our consortium [34]. It is not yet clear how the NK cell depletion occurs; RNAseq studies suggest enrichment of NK cells in the lungs [3, 85, 86], although in the absence of quantitative data, it is not clear whether this is due to migration from the periphery or *in situ* expansion of lung-resident NK cells.

## B CELLS

Robust antibody production has been widely detected in COVID-19 patients [87–91]. Accordingly, the percentage of antibody-producing plasmablasts has been reported to increase to 10–31% of circulating B cells [92, 93]. This can temporarily boost the WBC count, although the concurrent depletion of many other immune cell types, including CD19^+^ B cells [38, 57, 76, 78], seems to have largely masked this effect. It should also be noted that patients with severe COVID-19 can have high numbers of plasmablasts, suggesting that counts of these cells cannot be considered diagnostic without a qualitative analysis of the antibodies produced [28, 57, 92]. In line with this, higher amounts of IgM and IgG antibodies targeting SARS-CoV-2 nucleocapsid and spike proteins were associated with disease severity and poor clinical parameters [90, 94].

## T CELLS

The best-documented change in immune cells numbers in COVID-19 is in T cells, with 40–80% of patients showing T cell lymphopenia on admission [18, 20, 95], and a growing number of studies demonstrating a correlation between lymphopenia and severe COVID-19 [15, 18, 20, 75, 77, 95, 96]. Wang *et al.* found that T cell numbers are progressively reduced as the disease severity increases [77]. Peripheral T cell loss is associated with an increase in apoptotic cells and changes in the CXCR3 signalling pathway, suggesting that both T cell apoptosis and their migration towards inflamed tissue might contribute to peripheral lymphopenia [97]. Reduced lymphocyte count upon admission was found to not only increase disease severity but also mortality [78, 95, 98]. In particular, T cell counts <800/μl identify patients at risk of ICU admission and death [14, 78, 98]. Patients with lymphopenia also have an increased incidence of multi-organ injury, indicated by worse lung CT scores, reduced respiratory function and elevated indicators of hepatic injury [95].

Depletion of both CD4^+^ and CD8^+^ T cells has been reported in COVID-19 patients, in addition to phenotypic alterations in specific T cell subsets, which have recently been extensively reviewed by our consortium [99]. While some studies report preferential depletion of CD8^+^ T cells [92, 93], there is currently a lack of consensus as to the prognostic significance of reduced CD4^+^ and CD8^+^ T cell counts. Some studies have reported that reduction of each of these subsets is associated with COVID-19 severity and the need for ICU care [14, 76–78, 98]. However, in other reports, while both populations are decreased, only a reduction in one T cell subtype, CD4^+^ [15, 20, 100] or CD8^+^ [21, 101] is associated with disease severity or mortality.

A small number of studies have investigated the impact of SARS-CoV-2 infection on non-classical T cells. Mucosa-associated invariant T (MAIT) cells respond to cytokines, bacterial antigens and viruses [30, 102–104]. MAIT cells are depleted in COVID-19 patients [105] and this decrease is associated with disease severity [106, 108]. Flament *et al.* further suggest CD8^+^ MAIT cells were most prominently reduced, however, in contrast, Parrot *et al.* found that double negative (DN) MAIT were decreased to a greater degree than CD8^+^ MAIT [105, 106]. Invariant natural killer T (iNKT) cells are another innate T cell type, with cytotoxic, cytokine-producing and immunoregulatory roles, restricted by the recognition of lipid antigens [108]. Functional roles for iNKT cells have been described in the response to both chronic (HIV, hepatitis) and acute (influenza) viral infection [109], prompting their investigation in COVID-19. iNKT cells are found to be depleted in severe COVID-19 disease [28, 107].

There is little information as to the direct cause of T cell depletion in COVID-19. Reduction in CD4^+^ and CD8^+^ T cells also occurs in SARS-CoV-1 and MERS-CoV infections [110], however, only the latter has been shown to infect T cells and directly cause apoptosis [111]. Thus far there are no peer-reviewed data demonstrating SARS-CoV-2 infection of T cells. A study that used immortalized T cell lines to suggest cytotoxic SARS-CoV-2 infection of T cells has since been retracted [112]. Two studies have identified potential mechanisms by which T cells could be infected by SARS-CoV-2 [113, 114]. Pontelli *et al.* show that PBMCs, including CD4^+^ and CD8^+^ T cells, are susceptible to SARS-CoV-2 infection *in vitro,* as assessed by immunostaining of viral antigens and viral replication (dsRNA) [113]. The infection correlated with the expression of apoptotic markers, which may suggest a mechanism for lymphopenia in severe COVID-19 patients [113]. Wang *et al.* having previously demonstrated a role for CD147 in facilitating SARS-CoV-1 infection of target cells [115], showed that antibody blocking of CD147 inhibited SARS-CoV-2 infection of model cell lines (Vero E6 and BEAS-2B), and SARS-CoV-2 Spike gene expressing pseudovirus [116] infected CD4^+^ and CD8^+^ T cells [114]. However, more robust investigation of this interaction is required, since data of SARS-CoV-2 infection of T cells *in vivo* or *in-situ* is currently sparse. In post-mortem analysis of lung tissue, Carsana *et al.* found infected inflammatory monocytes, B and T cells, while SARS-CoV-2 infected macrophages have been identified in post-mortem spleen samples [31, 41]. Therefore, further investigation of SARS-CoV-2 infecting lymphoid organs is warranted.

Indirect factors are also likely to contribute to the T cell number reduction in COVID-19. Inflammation, particularly in severe patients, is well described [14, 15, 21, 40, 117] and levels of cytokines TNF, IL-6 and IL-10, and biochemical markers of inflammation negatively correlate with counts of CD4^+^ and CD8^+^ T cells [99, 101]. Increased TNF inflammation was observed post-mortem in secondary lymphoid organs and positively correlated with a loss in Bcl-6^+^ follicular helper T cells [118]. T cell numbers are also indirectly impacted by depletion and functional impairment of other immune cells. While more profound reduction in pDCs compared to cDCs is reported in COVID-19, cDCs from acute patients failed to respond to a maturation cocktail and did not simulate T cell proliferation [64]. Lack of cDC-mediated antigen presentation and co-stimulatory signals could therefore underlie low T cell counts. Depletion of NK cells negatively impacts signalling for DC maturation; additionally, NK cell cytokine production and co-stimulatory marker expression play a direct role in T cell differentiation [119]. Therefore, SARS-CoV-2 impairment of T cell responses involves multi-faceted disruption of the immune response. Furthermore, since enumeration of CD4^+^ and CD8^+^ T cell subsets in COVID-19 patients is not consistently accompanied by further in-depth phenotypic information, it is not clear whether reduced numbers of specific populations are due to conversions between particular phenotypes in the respective subset, or outright loss of cells [99, 120]*.* Reduced numbers of CD4^+^ and CD8^+^ T cells in COVID-19 patients may be due to failed activation/anergy, or hyperactivation, followed by apoptosis, each of which could contribute to severe COVID-19 [121].

## NEUTROPHILS AND THE NLR

As mentioned above, the decline in lymphocyte numbers combined with an increased neutrophil count, or NLR, has proven to be a strong predictor of disease severity and outcome [10, 18, 40, 122–124]. The NLR is indicative of a state of inflammation; neutrophils also impair T cell activation and proliferation [125, 126]. The NLR is prognostic in a large number of conditions including cancers, cardiovascular disease and infections [36, 127–133]. Meta-analysis has shown that the pooled risk ratio for mortality in COVID-19 patients with elevated NLR was 2.75 [124]. Rodriguez *et al.* additionally found that the NLR decreased with recovery [40].

The NLR also correlates with other immunological features of COVID-19. High NLR and anti-SARS-CoV-2 IgG antibody levels positively correlate with disease severity and negatively correlate with numbers of CD4^+^ T cells [134]. Depletion and functional impairment of NK cells may also exacerbate the NLR: IL-6-, IL-8- and IL-10-induced upregulation of the NKG2A inhibitory NK cell receptor inhibits the production of IFNγ, which controls neutrophil accumulation in the lung [135]. IL-6, which is widely reported to be increased in COVID-19 [17, 39], also directly regulates neutrophil trafficking [136]. In light of this, direct targeting of neutrophil associated COVID-19 pathology is under investigation in a number of clinical trials; blockade of neutrophil-driven complement signalling (Avdoralimab blockage of complement C5a: NCT04371367) and antibody inhibition of GM-CSF (Gimsilumab: NCT04351243), for which signalling is associated with neutrophilia [126]. These strategies are discussed in more detail in a complementary instalment of our review series [34]. Blockade of IL-6 signalling, using Tocilizumab, has also been investigated in COVID-19, and demonstrated patient discharge from the hospital within 28 days [137, 138].

## RESOLUTION OF THE IMMUNE PERTURBATIONS AND ASSOCIATED CLINICAL COURSE OF COVID-19

COVID-19 symptoms present at a median of 5.1 days from infection by SARS-CoV-2 [139], with a ‘tipping point’ towards worsening of the clinical condition and onset of ARDS occurring at 9–12 days from onset of symptoms [6, 43, 100, 140, 141]. Payen *et al.* describe a ‘V’ shaped curve of lymphocyte cell numbers, with the lowest point 11–14 days from symptom onset, i.e. overlapping with the point of symptomatic exacerbation [58].

Recovery of cell numbers to a normal range is generally associated with improvement in COVID-19. Longitudinal studies report a decrease in neutrophils and increase in basophils and eosinophils in the blood, prior to hospital discharge and in recovered patients; Mann *et al*. also noted that neutrophils increased from the first to final measures in *n* = 2 fatal COVID-19 cases [38, 40]. As neutrophilia is associated with COVID-19 severity, it is crucial to collect more longitudinal data to aid in-hospital treatment allocation and patient stratification. NK cell counts increased over the second to third week of observation in patients who had a favourable outcome, although higher NK cells at baseline were most commonly observed in mild cases [57]. DC numbers also increased between the symptom onset and recovery [40]. In individuals who experienced mild COVID-19, frequencies of monocytes and pDCs were comparable to healthy controls a median of 35 days after symptom onset; however, comparing frequencies may overlook a persistent overall reduction in absolute cell counts [142]. Inflammatory classical macrophages persist in the early recovery stage (ERS; <7 days since negative PCR test) [54], however, phenotypic rather than quantitative changes appear to be more relevant over the clinical course with regard to the different subsets of monocytes. Mann *et al.* report an increase in proliferating Ki67^+^ monocytes prior to ICU admission, suggesting that monocyte expansion contributes to disease exacerbation, potentially identifying a therapeutic target [38]. Payen *et al*. note that HLA-DR expression by monocytes was below the threshold defined for immunosuppression throughout the monitoring period, only recovering >24 days after initial symptoms. HLA-DR expression also positively correlated with absolute numbers of CD4^+^ and CD8^+^ T cells [58].

Immune modulating treatment is also likely to contribute to the improved numbers of innate immune cells and, by reducing innate immune-associated inflammation, clinical improvement observed in COVID-19 patients. However, the timing of treatment is crucial to avoid inhibition of the adaptive immune response, which is instrumental in resolving SARS-COV-2 infection [143]. For example, glucocorticoids induce neutrophil apoptosis but also inhibit DC function and T cell development [144]. Corticosteroid therapy has been administered to almost half of hospitalized COVID-19 patients (22–44.9%) [6, 21, 43, 54, 140, 145]. However, the rapidly evolving nature of the pandemic largely precludes in-depth longitudinal study of the effects of these treatments on quantitative immune cell recovery. The RECOVERY consortium concluded that dexamethasone and tocilizumab (an anti-IL-6 receptor antibody that increases IL-6 levels [45]), alone, or in combination, reduced mortality and hospital stay. However, this trial did not collect data pertaining to laboratory measures and therefore the effects on immune cell subsets cannot be ascertained [138, 146]. Additionally, a number of studies describe the improvement of innate cells, such as reduction in neutrophils and increase in non-classical and intermediate monocytes and DCs, in the absence of immunomodulatory treatment [40, 58].

While the data are still relatively limited, short-term (<30 days) monitoring of T cell counts in convalescent individuals suggests cell number recovery occurs on a trajectory dictated by disease severity. In two studies, T cell numbers began to recover in most mild patients within 10–15 days, while the duration of T cell recovery in moderate and severe patients lasted 5–20 days [38, 40]. Another study found that in severe patients, T cells recovered to numbers comparable to mild cases at 16 days after disease onset, although this was after a further depletion on Days 4–6 [21]. From the lowest point at 11–14 days, CD4^+^ and CD8^+^ T cell recovery in a cohort of ICU patients reached significance 19–23 days after ICU admission, although it was not clear if this time point took place under continued critical care [58]. T cell frequencies remained lower than in healthy controls in both early and late recovery stages, although these data were not stratified based on disease severity prior to the recovery stage [54]. However, impairment of polyfunctional responses, persistent activation and delayed quantitative recovery of IFNγ^+^ CD8^+^ T cells has also been identified [58, 147, 148].

It is not unexpected that recovery of T cell numbers takes longer in patients with more drastically depleted cell counts. Nevertheless, these trajectory data, along with the prognostic cell counts discussed in the previous sections, could be useful to predict the duration of patient’s critical care requirements. More information is also warranted on the recovery of T cells and other immune cell numbers, in comparison to healthy controls, to determine if this has any impact on the so-called ‘Long COVID’ syndrome of persistent, erratic symptoms which seem to occur irrespective of initial disease severity, and is currently largely characterized based on symptoms, without interrogation of the underlying physiology [149].

At the time of writing, two studies that have examined the recovery of adaptive immune cells up to 1, 3 and 8 months, report antigen-specific responses [142, 150]. Dan *et al.* report that 40–50% of their cohort had detectable SARS-CoV-2-specific CD4^+^ and CD8^+^ T cell responses at >6 months, although these were not enumerated [150]. Higher numbers of functional, Spike-specific memory B and CD4^+^ T cells were detected in response to *in vitro* stimulation at 1 and 3 months in recovered individuals [142]. Memory B cell increased, while memory CD4^+^ T cell remained similar (measured as counts or frequencies), from 1 to 3 months [142, 150]. Although lacking reinfection data, these studies encouragingly identify the capacity for reactivation of adaptive immunity against SARS-CoV-2, inviting further studies to determine if this response is protective.

## DISCUSSION

There are many factors not yet well understood in SARS-CoV-2 disease. Limitations of the available data include variation in study sizes, unevenly distributed or absent ethnicity data. In longitudinal studies, it is difficult to control for different numbers of data points per patient and the real-time nature of studies precludes uniform data collection time points. There is also an uneven distribution of data obtained from peripheral blood compared to BALF/lung tissue, leading to a lack of understanding of cell migration over the disease course, and the potential for under-reporting of an effective lung-resident immune response. Relative cell recovery from these different types of samples may also confound attempts to correlate cell numbers with clinical parameters.

Impairment of the immune response also goes beyond a simple depletion or expansion of immune cells. These metrics do however inform decision-making in the clinical setting based on patient stratification, particularly in the case of T cell lymphopenia, where counts can be used to stratify patients according to their need of intensive care facilities [14, 78, 98, 151]. Further analysis is required to determine at-risk lower limit cut-offs for NK cells, and more studies are required to reach a consensus upper limit of neutrophil counts and NLR. The prognostic value of other innate immune cells requires wider exploration of signalling, cytokine production and migration to the infection site, to determine the relationship between timing and efficacy in fighting SARS-CoV-2 infection. In particular, monocyte differentiation and phenotype proportions appear to correlate with disease severity, although this may prove ineffective as a prognostic indicator due to the complexity of monocyte characterization.

Due to the global impact of the SARS-CoV-2 pandemic, it is also important to consider the application of such metrics across the ‘normal ranges’ for diverse populations; for example, many of the studies reviewed here have a bias of sex or age range, due to the disproportionate impact of COVID-19 in some populations. Furthermore, it has been hypothesized that the worse survival rates reported in Black, Asian and minority ethnic backgrounds compared to white populations may be partly attributed to ‘underlying genetic factors’ [152]. However, most research papers, including many of the studies outlined here, have not specified the ethnicity of patients, or have analysed a relatively homogenous population, further limiting our understanding of why some people are at higher risk of adverse outcomes from COVID-19 infection [7].

## CONFLICT OF INTEREST STATEMENT

There is no conflict of interest for any of the authors.

Box 1: What is the consensus?

- Increased WBC counts occur in almost a quarter of COVID-19 patients and are associated with severe disease. However, WBC within physiological reference ranges may ‘mask’ a combination of extreme increases and decreases in the counts of different immune cells. Therefore, WBC count alone should not be used to stratify patients.
- An increase in neutrophils in the blood occurs in most COVID-19 patients. Both increased neutrophils and an increased NLR positively correlate with disease severity. Therapeutic modulation of neutrophil activation signalling is being tested in clinical trials, if approved this may provide a more targeted approach to reducing inflammation compared to corticosteroids which must be administered with appropriate timing so as not to inhibit the adaptive immune response.
- Both conventional and pDCs are reduced, the latter in association with severe disease. Cell counts do not sufficiently reveal the dysfunction of other monocytes; analysis of function is more likely to shed light on their contribution to COVID-19 pathology.
- Lymphopenia is the most widely reported immunopathological feature of COVID-19. This encompasses a reduction in NK cells, conventional αβ T cells and unconventional T cells including MAIT cells and iNKT cells. Total CD3^+^ T cell counts of <800/μl are predictive of a requirement for ICU care.
- While establishing the ‘starting point’ in COVID-19 is difficult (initial symptom onset vs. receipt of a positive test), the in-hospital disease course appears to segregate into stages. The biggest changes in cell counts and worsening of the clinical condition, including onset of ARDS, typically occur in the second week. Thereafter, cell numbers begin to recover, with cell numbers in more severe cases returning to normal ranges more slowly. Thus far, the limited data on long-term responses suggest B cell numbers are maintained for up to 3 months, while antigen-specific memory B and T cell responses following *in vitro* stimulation have been detected up to 8 months post COVID-19.

Box 2: Why does your reviewed topic matter in the pandemic?

- Cell counts are routinely measured making this an easily accessible resource that can be leveraged to inform patient prognosis and in-hospital care decisions.
- Changes in the number of immune cells in COVID-19 patients provide the first indicator of features of the immune response to SARS-CoV-2, to inform more in-depth research. Increased cell numbers can indicate effective activation and proliferation; however, in case of innate cells this is often delayed and, as a result, pathologically excessive in COVID-19. Decreased cell numbers, e.g. T cells, can occur due to a failure to provide the required co-stimulation or activation followed by apoptosis; these opposing responses could dictate a poor or effective immune response, respectively.

## AUTHOR CONTRIBUTIONS

A.M.G. and A.M. contributed to conceptualization and supervision; F.C.R. and D.O.S. contributed to project administration; A.S.C., E.B.C., F.C.R. and A.M. contributed to visualization; A.S.C., S.J.H., O.R.M., E.J.P., E.B.C., E.G.-M., M.B., A.M. and The Oxford-Cardiff COVID-19 Consortium contributed to writing—original draft; A.S.C., F.C.R. and A.M. contributed to revision; A.S.C., S.J.H., E.B.C., A.M. and F.C.R. contributed to writing—review and editing.

## DATA AVAILABILITY

All data are contained within the manuscript. This review was facilitated by weekly releases of the Oxford-Cardiff COVID-19 Literature Consortium journal cluba database of reviewed articles and journals will be made available on request.

## Appendix 1

David J. Ahern^1^, Hannah Almuttaqi^1^, Dominic S. Alonzi^2^, Aljawharah Alrubayyi^3^, Ghada Alsaleh^1^, Valentina M. T. Bart^4^, Vicky Batchelor^1^, Rebecca Bayliss^5^, Dorothée L. Berthold^1^, Jelena S. Bezbradica^1^, Tehmina Bharuchq^2^, Helene Borrmann^3^, Mariana Borsa^1^, Rowie Borst^1^, Juliane Brun^1^, Stephanie Burnell^4^, Lorenzo Capitani^4^, Athena Cavounidis^6^, Lucy Chapman^4^, Anne Chauveau^1^, Liliana Cifuentes^1^, Amy Susan Codd^4^, Ewoud Bernardus Compeer^1^, Clarissa Coveney^1^, Amy Cross^7^, Sara Danielli^1^, Luke C. Davies^4^, Calliope A. Dendrou^8^, Sandra Dimonte^4^, Ruban Rex Peter Durairaj^4^, Lynn B. Dustin^1^, Arthur Dyer^9^, Ceri Fielding^4^, Fabian Fischer^1^, Awen Gallimore^4^, Sarah Galloway^4^, Anís Gammage^1^, Ester Gea-Mallorquí^1^, Andrew Godkin^4^, Stephanie Jean Hanna^4^, Cornelia Heuberger^1^, Sarah Hulin-Curtis^4^, Fadi Issa^7^, Emma Jones^4^, Ruth Jones^10^, Kristin Ladell^4^, Sarah N. Lauder^4^, Kate Liddiard^5^, Petros Ligoxygakis^11^, Fangfang Lu^12^, Bruce MacLachlan^4^, Shayda Maleki-Toyserkani^4^, Elizabeth H. Mann^1^, Anna M. Marzeda^1^, Reginald James Matthews^13^, Julie M. Mazet^1^, Anita Milicic^14^, Emma Mitchell^4^, Owen Moon^4^, Van Dien Nguyen^4^, Miriam O'Hanlon^1^, Clara Eléonore Pavillet^18^, Dimitra Peppa^3^, Ana Pires^4^, Eleanor Pring^4^, Max Quastel^15^, Sophie Reed^4^, Jan Rehwinkel^16^, Niamh Richmond^1^, Felix Clemens Richter^1^, Alice J. B. Robinson^1^, Patrícia R. S. Rodrigues^4^, Pragati Sabberwal^4^, Arvind Sami^17^, Raphael Sanches Peres^1^, Quentin Sattentau^12^, Barbora Schonfeldova^1^, David Oliver Scourfield^4^, Tharini A. Selvakumar^15^, Freya R. Shepherd^4^, Cariad Shorten^18^, Anna Katharina Simon^1^, Adrian L. Smith^19^, Alicia Teijeira Crespo^5^, Michael Tellier^12^, Emily Thornton^16^, Lion F. K. Uhl^1^, Erinke van Grinsven^1^, Angus K. T. Wann^1^, Richard Williams^1^, Joseph D. Wilson^18^, Dingxi Zhou^1^, Zihan Zhu^12^

Affiliations of Consortium members: ^1^Nuffield Department of Orthopaedics, Rheumatology and Musculoskeletal Sciences, Kennedy Institute of Rheumatology, University of Oxford, Oxford, UK, ^2^Department of Biochemistry, Oxford Glycobiology Institute, University of Oxford, Oxford, UK, ^3^Nuffield Department of Clinical Medicine, Nuffield Department of Medicine, University of Oxford, Oxford, UK, ^4^Division of Infection and Immunity, School of Medicine, Cardiff University, Cardiff, UK, ^5^Division of Cancer and Genetics, School of Medicine, Cardiff University, Cardiff, UK, ^6^Translational Gastroenterology Unit, Nuffield Department of Medicine, University of Oxford, Oxford, UK, ^7^Nuffield Department of Surgical Sciences, University of Oxford, Oxford, UK, ^8^Wellcome Centre for Human Genetics, Nuffield Department of Medicine, University of Oxford, Oxford, UK, ^9^Department of Oncology, University of Oxford, Oxford, UK, ^10^Dementia Research Institute, Haydn Ellis Building, Cardiff University, Cardiff, UK, ^11^Department of Biochemistry, University of Oxford, Oxford, UK, ^12^Sir William Dunn School of Pathology, Medical Science Division, University of Oxford, Oxford, UK, ^13^Centre for Medical Education, School of Medicine, Cardiff University, Cardiff, UK, ^14^The Jenner Institute, Nuffield Department of Medicine, University of Oxford, Oxford, UK, ^15^Nuffield Department of Medicine, University of Oxford, Oxford, UK, ^16^Medical Research Council Human Immunology Unit, Medical Research Council Weatherall Institute of Molecular Medicine, Radcliffe Department of Medicine, University of Oxford, Oxford, UK, ^17^Botnar Research Centre, Nuffield Department of Orthopaedics, Rheumatology and Musculoskeletal Sciences, University of Oxford, Oxford, UK, ^18^Medical Sciences Division, University of Oxford, Oxford, UK, ^19^Department of Zoology, University of Oxford, Oxford, UK
